# Bis(di­allyl­carbamodi­thio­ato)(quinaldine)zinc(II)

**DOI:** 10.1107/S2056989026007206

**Published:** 2026-07-21

**Authors:** Mihai Raduca, Sergiu Shova, Florea Dumitrascu, Constantin Draghici, Madalina Hrubaru

**Affiliations:** a C. D. Nenitzescu Institute of Organic and Supramolecular Chemistry, Romanian Academy, 202B Splaiul Independentei, 060023 Bucharest, Romania; b Department of Inorganic Polymers, Petru Poni Institute of Macromolecular Chemistry, Aleea Grigore Ghica Voda nr. 41A, 700487 Iasi, Romania; Tokyo University of Science, Japan

**Keywords:** crystal structure, zinc, 2-methyl­quinoline, di­allyl­carbamodi­thio­ate, quinaldine

## Abstract

The crystal structure of the title compound {systematic name: [bis(pro­p-2-en-1-yl)car­bam­o­di­thio­ato-κ^2^*S*,*S*′](2-methyl­quino­line-κ*N*)zinc(II)}, [Zn(C_7_H_10_NS_2_)_2_(C_10_H_9_N)] the heteroleptic complex reveals supra­molecular dimers established by π–π inter­actions between adjacent quinaldine units with a centroid–centroid distance of 3.672 (2) Å. The compound crystallizes in the triclinic crystal system, space group *P*1, with the Zn^II^ atom exhibiting a trigonal–bipyramidal geometry.

## Abstract 2

The crystal structure of the title compound, the heteroleptic complex [Zn(C_7_H_10_NS_2_)_2_(C_10_H_9_N)] reveals supra­molecular dimers established by π–π inter­actions between adjacent quinaldine units with a centroid–centroid distance of 3.672 (2) Å. The compound crystallizes in the triclinic crystal system, space group *P*1, with the Zn^II^ atom exhibiting a trigonal–bipyramidal geometry.

## 1. Chemical context

Metal complexes of di­thio­carbamate and its substituted deriv­atives are known for their diverse catalytic functions and promising biological activities (Thammakan & Somsook, 2006 ▸). Among di­thio­carbamate complexes, zinc(II) derivatives have received considerable attention as anti­oxidant additives for plastics and hydro­carbon-based lubricants, with studies examining their mechanisms of action (Ali *et al.*, 2006 ▸). Moreover, quinaldine is a versatile inter­mediate for the synthesis of pharmaceuticals, dyes, agrochemicals, and fine chemicals (Matada *et al.*, 2021 ▸). Its derivatives often display biological activities, including anti­microbial, anti-inflammatory, and anti­cancer potential, making them valuable targets in medicinal chemistry research (Yadav & Shah, 2021 ▸). Numerous structurally characterized zinc(II) di­thio­carbamate complexes containing N-heterocyclic ligands have been reported in the literature; however, no analogous complex incorporating quinaldine has been described to date (Reck & Becker, 2004*a* ▸). Here we describe the crystal structure of a mononuclear heteroleptic Zn^II^ complex bearing di­allyl­dithio­carbamate (*L*^−^) and 2-methyl­quinoline (Qy) ligands (**1**).
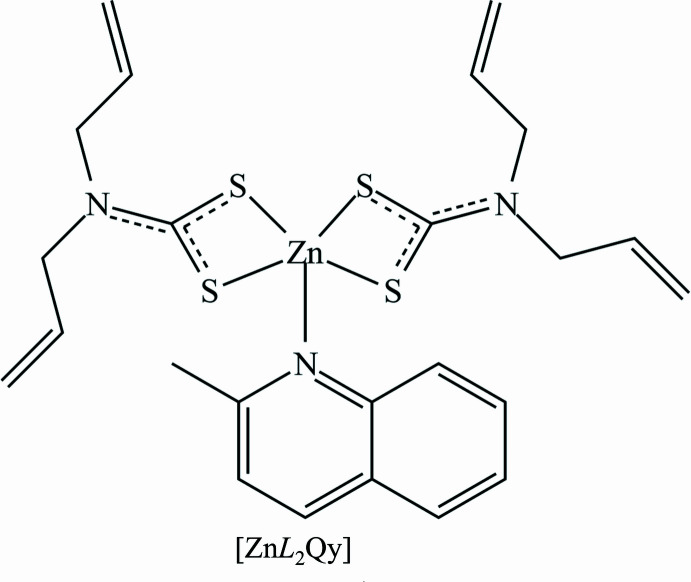


## 2. Structural commentary

**Figure 1:**
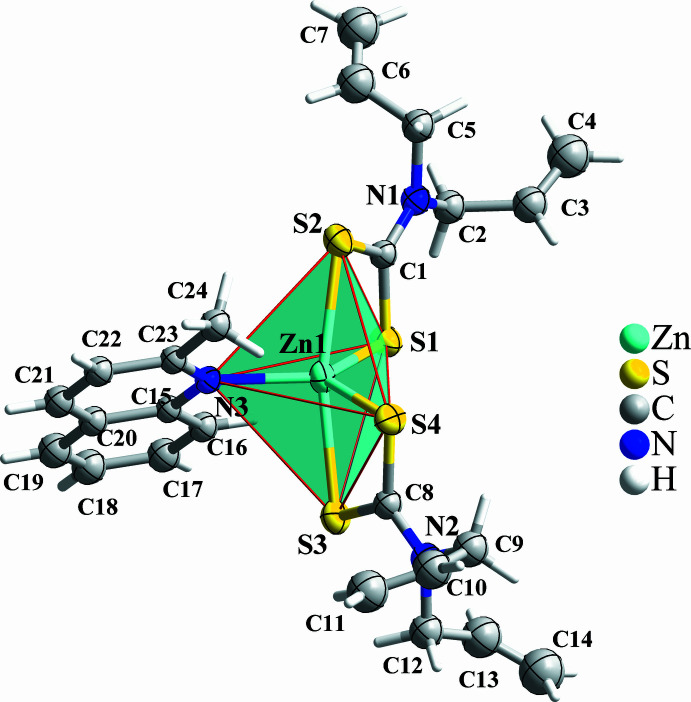
The mol­ecular structure of the title compound **1**, showing the trigonal–bipyramidal coordination geometry around the Zn^II^ atom in the first coordination sphere. Displacement ellipsoids are drawn at the 50% probability level.

Although Zn^II^ is stereochemically a labile ion and its coordination chemistry is therefore governed more by ligand architecture than by the electronic stabilization, the most frequent coordination numbers are four and six (Melnik *et al.*, 1995 ▸). When analysing five-coordinate zinc complexes, generally two principal geometries occur: square pyramidal and trigonal bipyramidal. The coordination environment of the Zn^II^ cation in compound **1** is observed to have a distorted trigonal–bipiramidal geometry (SHAPE analysis: TBPY-5 = 1.996) in which the basal plane is described by the nitro­gen atom of the quinaldine ligand and one of the sulfur atoms from the two thio­carboxyl­ate units (Fig. 1 ▸). The Zn^II^ cation is coordinated by four S atoms with distances ranging from 2.3669 (9) to 2.6080 (10) Å and an N atom at a distance of 2.103 (2) Å. The C—N bond length to the di­thio­carbamate moiety is 1.331 (4) Å. These bond lengths are similar to those reported for related complexes (see Section 4: *Database survey*). Intra­molecular short contacts and hydrogen bonds (Table 1 ▸) are observed.

## 3. Supra­molecular features

**Figure 2:**
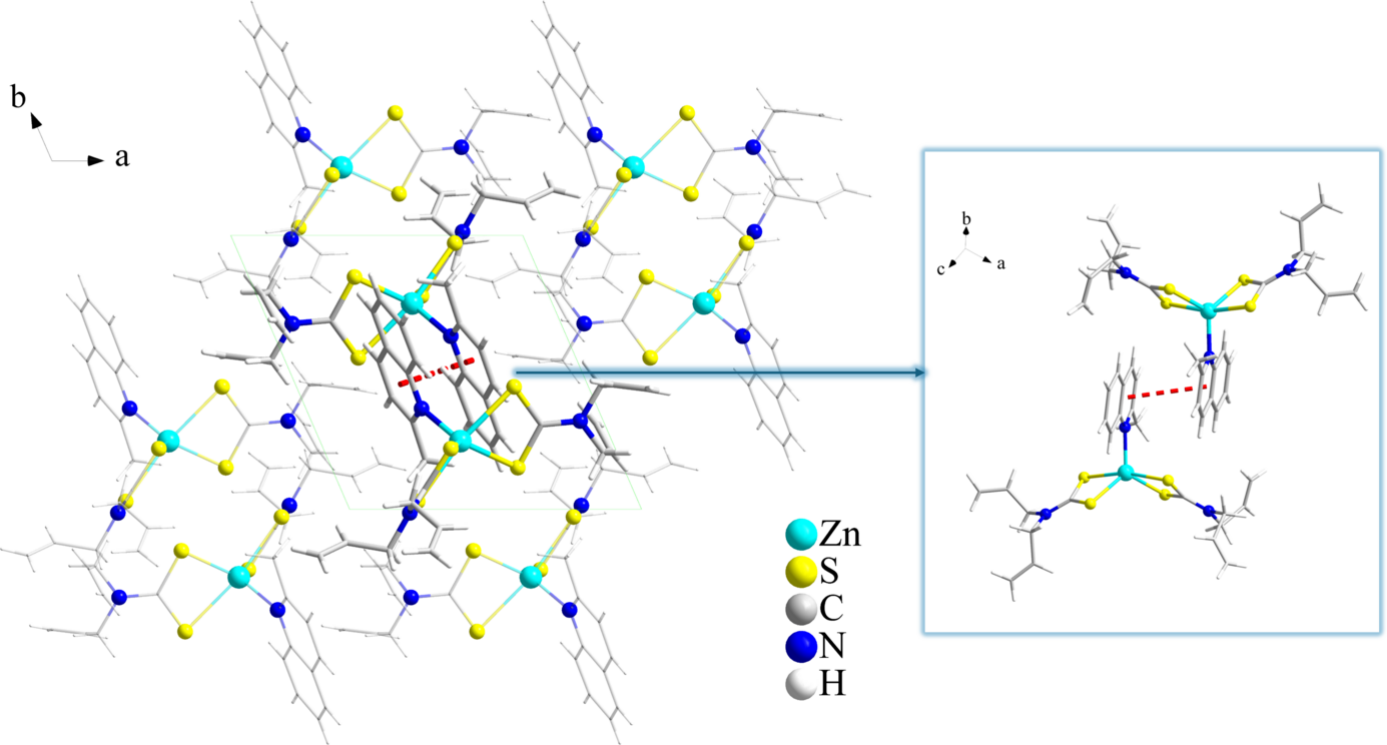
View along the *c* axis of the packing of compound **1** showing π–π stacking between neighbouring quinaldine mol­ecules. Insert: detail of π–π inter­actions between quinaldine units, *Cg*⋯*Cg* = 3.672 (2) Å.

The packing of **1** reveals π–π inter­actions between adjacent heterocycles of quinaldine mol­ecules [inter­centroid distance = 3.672 (2) Å], forming dimers (Fig. 2 ▸).

## 4. Database survey

A survey of the Cambridge Structural Database (CSD, version6.01 February 2026; Groom *et al.*, 2016 ▸) showed more than 200 complexes in which Zn^II^ atoms are coordinated by four sulfur atoms and one nitro­gen. More than 170 structures exhibit a ZnS_4_N coordination environment with the four sulfur atoms belonging to two thio­carbamato units. Among them, around 50 examples crystallize in the triclinic crystal system. Although there are examples in which there is another coordinating atom, usually nitro­gen, which ensures a coordination number of 6 (Reck & Becker, 2004*b* ▸; Srinivasan *et al.*, 2013 ▸; Ramalingam *et al.*, 2010 ▸), the majority of the structures exhibit coordination number 5 where the nitro­gen atom belongs to a ligand such as pyridine (Selvaganapathi *et al.*, 2018 ▸; Jamuna Rani *et al.*, 2015 ▸; Dulare *et al.*, 2012 ▸; Malik *et al.*, 1997 ▸), pyrazine (Jotani *et al.*, 2017 ▸), imidazole (Chen & Powers, 1995 ▸), urotropine (Konarev *et al.*, 2008 ▸; Câmpian *et al.*, 2016 ▸) or 4,4′-bi­pyridine (Manohar *et al.*, 2001 ▸; Benson *et al.*, 2007 ▸). The coordinative bond lengths for the similar complexes are listed in Table 2 ▸.

## 5. Synthesis and crystallization

All solvents were of analytical grade and were used without further purification. The metal salt and the reagents used in the ligand synthesis were obtained from commercial sources. For the synthesis of the Zn di­allyl­dithio­carbamate complex [Zn*L*_2_], the previously reported procedure was used (Hrubaru *et al.*, 2016 ▸). After recrystallization from chloro­form, the [Zn*L*_2_] complex was further used for the synthesis of the corresponding adduct with 2-quinaldine.

The [Zn*L*_2_Qy] adduct was also prepared following a previously reported method (Onwudiwe *et al.*, 2016 ▸). Thus, approximately 0.3 g of [Zn*L*_2_] were dissolved in 10 mL of quinaldine solution (Lewis base), and the resulting suspension was refluxed for 8 h at a temperature between 363–373 K. After a while the solution became clear, and shortly thereafter a white precipitate began to form. The reaction was allowed to cool, and the quinaldine solution was filtered. The precipitate obtained was rinsed with water, followed by ethanol, and recrystallized from hot chloro­form. Yield: 0.38 g, 56%, m.p.: 379–382 K.
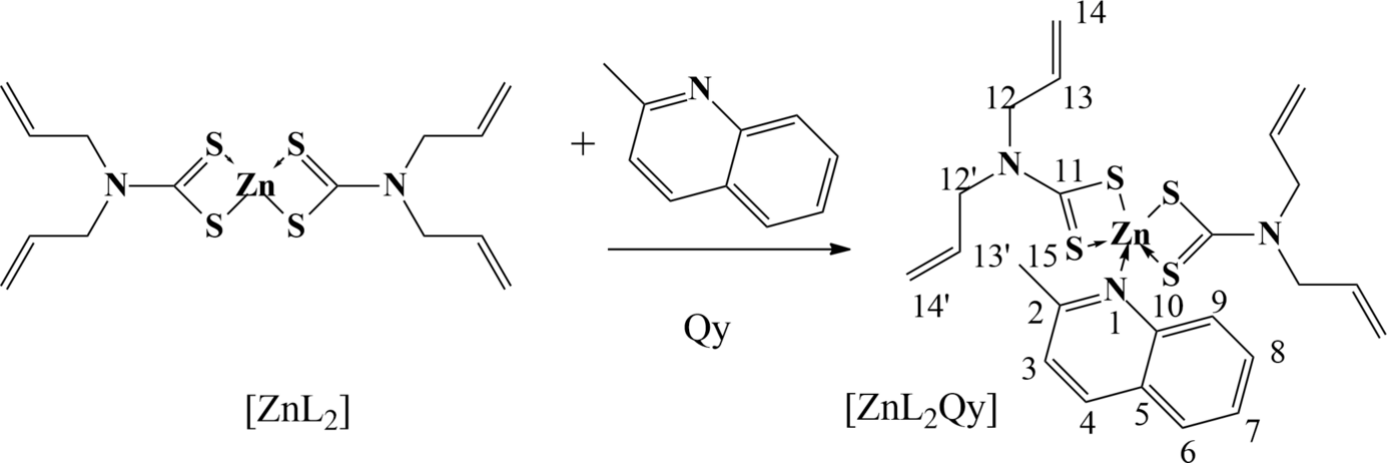


^1^H-NMR and ^13^C-NMR spectra were recorded on Varian Mercury Plus 300 MHz spectrometer equipped with Probe 300AutoSw PFG 4 NUC/30-122 MHz; the chemical shifts are given in ppm relative to TMS as inter­nal standard. Complementary spectra: 2D-NMR were done for the correct assignment of NMR signals. The chemical shifts δ are expressed in ppm (δ) and the coupling constants *J* in Hz. The following abbreviations were used to characterize the signals: *s* – singlet, *d* – doublet, *t* – triplet, *m* – multiplet, *ddd* – double doublet, *td* – triple doublet, *bd* – broadened doublet. Infrared (solid ATR) spectra were recorded on a FT-IR Bruker Vertex 70 by ATR spectrometer directly on small samples of the compounds in the range of 4000–400 cm^−1^. The following abbreviations were used to characterize the signals: *w* = weak, *m* = medium, *s* = strong, *v* = very, *br* = broad.

^1^H-NMR (CDCl_3_, δ ppm, *J* Hz): 8.03 (*d*, 8.2, 1H, H3), 8.01 [*bd*, 7.5, 1H, H9(6)], 7.27 (*d*, 8.2, 1H, H4), 7.76 [*dd*, 7.9, 1.5, 1H, H6(9)], 7.67 [*td*, 7.0, 1.4, 1H, H-7(8)], 7.46 [*td*, 7.0, 1.4, 1H, H8(7)], 7.27 (*d*, 8.2, 1H, H3), 5.86 (*ddt*, 17.0, 10.2, 5.9, 1H, H13), 4.45 (*bd*, 5.9, 2H, H12); 2.74 (*s*, H15), 5.28 (*bd*, 10.2, H14*cis*), 5.24 (*bd*, 17.0, H14*trans*). ^13^C-NMR (CDCl_3_, δ ppm): 205.2 (C11), 159.1(C2), 147.9 (C10), 136.3 (C4), 130.3 (C13), 129.5 (C8), 128.7 (C9), 127.6 (C6), 126.6 (C5), 125.8 (C7), 122.1 (C3), 119.5 (C14), 56.1 (C12), 25.5 (C15). Selected ν IR data (solid ATR, cm^−1^): 3075.3*w*, 2974.4*w*, 2918.9*w*, 1638.7*w*, 1486.3*vs*, 1464.8*vs*, 1426.5*s*, 1405.7*vs*, 1339.6, 1276.8*w*, 1230.6*vs*, 1173.6*s*, 1100.6*m*, 989.3*s*, 928.91*s*, 915.7*vs*, 682.7*w*, 651.2*w*, 577.3*w*, 545.4*w*. Crystals suitable for single-crystal X-ray analysis were obtained by recrystallization from chloro­form/ethanol (50/50 *v*/*v*).

## 6. Refinement

Crystal data, data collection and structure refinement details are summarized in Table 3 ▸. H atoms were positioned geometrically and were allowed to ride on the parent C atoms and rotate around C—C bonds.

## Supplementary Material

Crystal structure: contains datablock(s) I, my_structure. DOI: 10.1107/S2056989026007206/jp2029sup1.cif

Structure factors: contains datablock(s) I. DOI: 10.1107/S2056989026007206/jp2029Isup2.hkl

CCDC reference: 2556564

Additional supporting information:  crystallographic information; 3D view; checkCIF report

## Figures and Tables

**Table 1:** Hydrogen-bond geometry (Å, °)

*D*—H⋯*A*	*D*—H	H⋯*A*	*D*⋯*A*	*D*—H⋯*A*
C2—H2*B*⋯S1	0.97	2.55	3.048 (4)	112
C9—H9*A*⋯S4	0.97	2.52	2.999 (4)	110
C16—H16⋯S1	0.93	2.88	3.742 (3)	155
C24—H24*A*⋯S4	0.96	2.78	3.694 (3)	158
C24—H24*C*⋯S2	0.96	2.90	3.505 (4)	122

**Table 2:** The coordination bond lengths (Å) in **1** compared with those in similar complexes

Refcode	CCDC No.	Zn1—S	Zn1—N	Reference
MIDQEY	1584489	2.3569 (10)–2.6047 (10)	2.068 (3)	Selvaganapathi *et al.* (2018 ▸)
NORBIH	948010	2.3574 (6)–2.6200 (6)	2.0579 (17)	Jamuna Rani *et al.* (2015 ▸)
RALQEC	773588	2.3770 (15)–2.5251 (13)	2.069 (4)	Dulare *et al.* (2012 ▸)
RIPKUW	1250327	2.3371 (4)–2.6124 (5)	2.069 (3)	Malik *et al.* (1997 ▸)
QEVDAZ	1507480	2.4121 (6)–2.4629 (6)	2.1419 (13)	Jotani *et al.* (2017 ▸)
TEDVON	1268607	2.3622 (4)–2.5816 (4)	2.0086 (11)	Chen & Powers, 1995 ▸)
QIYVEA01	638246	2.3533 (6)–2.6257 (6)	2.1207 (18)	Konarev *et al.* (2008 ▸)
YAKYOB	1409585	2.3358 (7)–2.6525 (8)	2.102 (2)	Câmpian *et al.* (2016 ▸)
XEYWIH	147187	2.3487 (2)–2.6019 (2)	2.0721 (5)	Manohar *et al.* (2001 ▸)
XEYWIH01	645547	2.3628 (12)–2.6074 (13)	2.082 (3)	Benson *et al.* (2007 ▸)
Compound **1**	2558105	2.3669 (9)–2.6080 (10)	2.103 (2)	This work

**Table 3:** Experimental details

Crystal data	
Chemical formula	[Zn(C_7_H_10_NS_2_)_2_(C_10_H_9_N)]
*M*_r_	553.11
Crystal system, space group	Triclinic, *P* 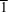
Temperature (K)	199
*a*, *b*, *c* (Å)	10.1713 (8), 10.2868 (6), 14.1190 (9)
α, β, γ (°)	97.659 (5), 101.518 (6), 111.145 (6)
*V* (Å^3^)	1315.54 (17)
*Z*	2
Radiation type	Mo *K*α
μ (mm^−1^)	1.27
Crystal size (mm)	0.2 × 0.2 × 0.15

Data collection	
Diffractometer	Xcalibur, Eos
Absorption correction	Multi-scan (*CrysAlis PRO*; Rigaku OD, 2015 ▸)
*T*_min_, *T*_max_	0.705, 1.000
No. of measured, independent and observed [*I* > 2σ(*I*)] reflections	10083, 4641, 3738
*R*_int_	0.038
(sin θ/λ)_max_ (Å^−1^)	0.595

Refinement	
*R*[*F*^2^ > 2σ(*F*^2^)], *wR*(*F*^2^), *S*	0.043, 0.106, 1.01
No. of reflections	4641
No. of parameters	290
H-atom treatment	H-atom parameters constrained
Δρ_max_, Δρ_min_ (e Å^−3^)	0.60, −0.32

Computer programs: *CrysAlis PRO* (Rigaku OD, 2015 ▸), *SHELXT* (Sheldrick, 2015*a* ▸), *SHELXL2018/3* (Sheldrick, 2015*b* ▸) and *OLEX2* (Dolomanov *et al.*, 2009 ▸).

## supplementary crystallographic information

### [Bis(prop-2-en-1-yl)carbamodithioato-κ2S,S'](2-methylquinoline-κN)zinc(II) Crystal data

**Table:**

[Zn(C_7_H_10_NS_2_)_2_(C_10_H_9_N)]	*Z* = 2
*M**_r_* = 553.11	*F*(000) = 576
Triclinic, *P*1	*D*_x_ = 1.396 Mg m^−^^3^
*a* = 10.1713 (8) Å	Mo *K*α radiation, λ = 0.71073 Å
*b* = 10.2868 (6) Å	Cell parameters from 3806 reflections
*c* = 14.1190 (9) Å	θ = 1.5–31.6°
α = 97.659 (5)°	µ = 1.27 mm^−^^1^
β = 101.518 (6)°	*T* = 199 K
γ = 111.145 (6)°	Prism, clear light colourless
*V* = 1315.54 (17) Å^3^	0.2 × 0.2 × 0.15 mm

### [Bis(prop-2-en-1-yl)carbamodithioato-κ2S,S'](2-methylquinoline-κN)zinc(II) Data collection

**Table:**

Xcalibur, Eos diffractometer	4641 independent reflections
Radiation source: fine-focus sealed X-ray tube, Enhance (Mo) X-ray Source	3738 reflections with *I* > 2σ(*I*)
Graphite monochromator	*R*_int_ = 0.038
Detector resolution: 16.1593 pixels mm^-1^	θ_max_ = 25.0°, θ_min_ = 2.2°
ω scans	*h* = −12→12
Absorption correction: multi-scan (CrysAlisPro; Rigaku OD, 2015)	*k* = −12→12
*T*_min_ = 0.705, *T*_max_ = 1.000	*l* = −16→16
10083 measured reflections	

### [Bis(prop-2-en-1-yl)carbamodithioato-κ2S,S'](2-methylquinoline-κN)zinc(II) Refinement

**Table:**

Refinement on *F*^2^	Primary atom site location: dual
Least-squares matrix: full	Hydrogen site location: inferred from neighbouring sites
*R*[*F*^2^ > 2σ(*F*^2^)] = 0.043	H-atom parameters constrained
*wR*(*F*^2^) = 0.106	*w* = 1/[σ^2^(*F*_o_^2^) + (0.0513*P*)^2^] where *P* = (*F*_o_^2^ + 2*F*_c_^2^)/3
*S* = 1.01	(Δ/σ)_max_ < 0.001
4641 reflections	Δρ_max_ = 0.60 e Å^−^^3^
290 parameters	Δρ_min_ = −0.32 e Å^−^^3^
0 restraints	

### [Bis(prop-2-en-1-yl)carbamodithioato-κ2S,S'](2-methylquinoline-κN)zinc(II) Special details

**Table:**

Geometry. All esds (except the esd in the dihedral angle between two l.s. planes) are estimated using the full covariance matrix. The cell esds are taken into account individually in the estimation of esds in distances, angles and torsion angles; correlations between esds in cell parameters are only used when they are defined by crystal symmetry. An approximate (isotropic) treatment of cell esds is used for estimating esds involving l.s. planes.
Refinement. The structure was solved with ShelXT program using intrinsic phasing method and refined by full-matrix least squares method on F^2^ with ShelXL (Sheldrick, 2008, 2015b,a). Olex2 was used as an interface to the ShelX program (Dolomanov *et al.*, 2009). All non-H atoms were refined anisotropically. H atoms were positioned geometrically and refined with riding coordinates. CH_3_ H atoms were positioned geometrically and were allowed to ride on C atom and rotate around C-C bond. Empirical absorption correction using spherical harmonics was applied.

### [Bis(prop-2-en-1-yl)carbamodithioato-κ2S,S'](2-methylquinoline-κN)zinc(II) Fractional atomic coordinates and isotropic or equivalent isotropic displacement parameters (Å^2^)

**Table:**

	*x*	*y*	*z*	*U*_iso_*/*U*_eq_	
Zn1	0.48037 (4)	0.24861 (4)	0.21013 (3)	0.03032 (14)	
S1	0.43656 (9)	0.22107 (9)	0.36779 (6)	0.0361 (2)	
S2	0.24447 (10)	0.02821 (9)	0.17742 (6)	0.0355 (2)	
S3	0.74518 (10)	0.44703 (9)	0.27158 (6)	0.0357 (2)	
S4	0.63785 (9)	0.15174 (9)	0.15520 (6)	0.0362 (2)	
N1	0.2005 (3)	−0.0136 (3)	0.35258 (18)	0.0330 (6)	
N2	0.9210 (3)	0.3231 (3)	0.2269 (2)	0.0346 (6)	
N3	0.3965 (3)	0.3680 (3)	0.12491 (18)	0.0276 (6)	
C1	0.2835 (3)	0.0695 (3)	0.3044 (2)	0.0284 (7)	
C2	0.2210 (4)	0.0235 (4)	0.4605 (2)	0.0400 (8)	
H2A	0.127682	0.011472	0.473488	0.048*	
H2B	0.286981	0.123159	0.486343	0.048*	
C3	0.2816 (4)	−0.0674 (4)	0.5122 (3)	0.0497 (10)	
H3	0.372719	−0.063582	0.507407	0.060*	
C4	0.2153 (5)	−0.1515 (5)	0.5636 (3)	0.0707 (13)	
H4A	0.124016	−0.157600	0.569730	0.085*	
H4B	0.258823	−0.205888	0.594341	0.085*	
C5	0.0732 (4)	−0.1484 (3)	0.3007 (3)	0.0405 (9)	
H5A	0.089990	−0.189264	0.240548	0.049*	
H5B	0.061618	−0.216608	0.342888	0.049*	
C6	−0.0607 (4)	−0.1217 (4)	0.2755 (3)	0.0562 (11)	
H6	−0.064808	−0.064191	0.230457	0.067*	
C7	−0.1717 (5)	−0.1695 (4)	0.3093 (3)	0.0666 (12)	
H7A	−0.172897	−0.227490	0.354568	0.080*	
H7B	−0.251549	−0.146342	0.288643	0.080*	
C8	0.7830 (4)	0.3092 (3)	0.2187 (2)	0.0306 (7)	
C9	0.9546 (4)	0.2061 (4)	0.1823 (3)	0.0462 (10)	
H9A	0.873727	0.116015	0.175816	0.055*	
H9B	1.040607	0.205626	0.226569	0.055*	
C10	0.9815 (4)	0.2158 (4)	0.0836 (3)	0.0570 (11)	
H10	1.007909	0.146333	0.053686	0.068*	
C11	0.9717 (4)	0.3117 (6)	0.0348 (3)	0.0705 (14)	
H11A	0.945643	0.383412	0.061617	0.085*	
H11B	0.990827	0.308745	−0.026997	0.085*	
C12	1.0473 (4)	0.4538 (4)	0.2830 (3)	0.0444 (9)	
H12A	1.025038	0.536334	0.274424	0.053*	
H12B	1.130480	0.461079	0.257136	0.053*	
C13	1.0861 (5)	0.4548 (5)	0.3910 (3)	0.0650 (13)	
H13	1.022201	0.467706	0.426497	0.078*	
C14	1.1978 (5)	0.4398 (5)	0.4396 (3)	0.0789 (15)	
H14A	1.265039	0.426548	0.407537	0.095*	
H14B	1.212019	0.442023	0.507039	0.095*	
C15	0.4040 (3)	0.5001 (3)	0.1689 (2)	0.0258 (7)	
C16	0.4472 (3)	0.5463 (3)	0.2726 (2)	0.0310 (7)	
H16	0.472382	0.488546	0.311754	0.037*	
C17	0.4526 (4)	0.6761 (4)	0.3167 (3)	0.0404 (9)	
H17	0.480095	0.704657	0.385486	0.048*	
C18	0.4172 (4)	0.7663 (4)	0.2592 (3)	0.0434 (9)	
H18	0.421761	0.854212	0.289847	0.052*	
C19	0.3764 (4)	0.7244 (4)	0.1588 (3)	0.0419 (9)	
H19	0.353669	0.784583	0.120956	0.050*	
C20	0.3678 (3)	0.5908 (3)	0.1109 (2)	0.0312 (7)	
C21	0.3223 (4)	0.5421 (4)	0.0069 (2)	0.0362 (8)	
H21	0.299591	0.599907	−0.033241	0.043*	
C22	0.3119 (3)	0.4110 (4)	−0.0345 (2)	0.0340 (8)	
H22	0.280925	0.378382	−0.103070	0.041*	
C23	0.3476 (3)	0.3238 (3)	0.0260 (2)	0.0280 (7)	
C24	0.3329 (4)	0.1782 (3)	−0.0220 (2)	0.0376 (8)	
H24A	0.418591	0.163390	0.007219	0.056*	
H24B	0.322562	0.171419	−0.091759	0.056*	
H24C	0.248210	0.106698	−0.012062	0.056*	

### [Bis(prop-2-en-1-yl)carbamodithioato-κ2S,S'](2-methylquinoline-κN)zinc(II) Atomic displacement parameters (Å^2^)

**Table:**

	*U*^11^	*U*^22^	*U*^33^	*U*^12^	*U*^13^	*U*^23^
Zn1	0.0368 (2)	0.0322 (2)	0.0286 (2)	0.01821 (19)	0.01148 (17)	0.01121 (17)
S1	0.0402 (5)	0.0348 (5)	0.0288 (4)	0.0099 (4)	0.0083 (4)	0.0083 (4)
S2	0.0445 (5)	0.0341 (5)	0.0259 (4)	0.0139 (4)	0.0084 (4)	0.0073 (4)
S3	0.0422 (5)	0.0314 (5)	0.0365 (5)	0.0190 (4)	0.0108 (4)	0.0048 (4)
S4	0.0367 (5)	0.0306 (5)	0.0409 (5)	0.0149 (4)	0.0098 (4)	0.0030 (4)
N1	0.0393 (16)	0.0263 (15)	0.0281 (14)	0.0091 (13)	0.0057 (12)	0.0054 (12)
N2	0.0362 (16)	0.0288 (15)	0.0420 (16)	0.0153 (13)	0.0142 (13)	0.0067 (13)
N3	0.0302 (14)	0.0285 (14)	0.0282 (14)	0.0138 (12)	0.0086 (11)	0.0120 (12)
C1	0.0377 (18)	0.0253 (17)	0.0290 (16)	0.0189 (15)	0.0093 (14)	0.0092 (14)
C2	0.046 (2)	0.041 (2)	0.0272 (17)	0.0094 (18)	0.0122 (16)	0.0060 (16)
C3	0.044 (2)	0.072 (3)	0.038 (2)	0.026 (2)	0.0085 (17)	0.020 (2)
C4	0.074 (3)	0.086 (3)	0.066 (3)	0.039 (3)	0.017 (2)	0.043 (3)
C5	0.049 (2)	0.0292 (19)	0.0370 (19)	0.0085 (17)	0.0103 (17)	0.0098 (16)
C6	0.045 (2)	0.037 (2)	0.065 (3)	0.002 (2)	−0.002 (2)	0.013 (2)
C7	0.061 (3)	0.054 (3)	0.066 (3)	0.012 (2)	0.008 (2)	−0.004 (2)
C8	0.042 (2)	0.0303 (18)	0.0249 (16)	0.0177 (16)	0.0110 (14)	0.0121 (14)
C9	0.037 (2)	0.037 (2)	0.071 (3)	0.0229 (18)	0.0127 (19)	0.0088 (19)
C10	0.044 (2)	0.056 (3)	0.063 (3)	0.018 (2)	0.018 (2)	−0.012 (2)
C11	0.045 (3)	0.109 (4)	0.051 (3)	0.025 (3)	0.018 (2)	0.007 (3)
C12	0.036 (2)	0.040 (2)	0.053 (2)	0.0107 (18)	0.0147 (18)	0.0052 (18)
C13	0.051 (3)	0.082 (3)	0.049 (2)	0.018 (2)	0.014 (2)	−0.008 (2)
C14	0.064 (3)	0.117 (4)	0.053 (3)	0.030 (3)	0.020 (2)	0.023 (3)
C15	0.0231 (16)	0.0253 (17)	0.0307 (16)	0.0103 (14)	0.0085 (13)	0.0081 (14)
C16	0.0322 (18)	0.0290 (18)	0.0322 (17)	0.0138 (15)	0.0064 (14)	0.0068 (15)
C17	0.036 (2)	0.042 (2)	0.0390 (19)	0.0161 (17)	0.0059 (16)	−0.0002 (17)
C18	0.042 (2)	0.033 (2)	0.058 (2)	0.0216 (18)	0.0115 (18)	0.0039 (18)
C19	0.045 (2)	0.038 (2)	0.050 (2)	0.0242 (18)	0.0123 (18)	0.0141 (18)
C20	0.0272 (17)	0.0304 (18)	0.0411 (19)	0.0139 (15)	0.0114 (15)	0.0141 (15)
C21	0.0345 (19)	0.044 (2)	0.0374 (18)	0.0211 (17)	0.0075 (15)	0.0206 (16)
C22	0.0293 (18)	0.044 (2)	0.0284 (17)	0.0142 (16)	0.0061 (14)	0.0119 (16)
C23	0.0237 (16)	0.0319 (18)	0.0275 (16)	0.0090 (15)	0.0080 (13)	0.0079 (14)
C24	0.047 (2)	0.0326 (19)	0.0263 (16)	0.0128 (17)	0.0048 (15)	0.0021 (15)

### [Bis(prop-2-en-1-yl)carbamodithioato-κ2S,S'](2-methylquinoline-κN)zinc(II) Geometric parameters (Å, º)

**Table:**

Zn1—S1	2.3888 (9)	C9—H9B	0.9700
Zn1—S2	2.5425 (10)	C9—C10	1.482 (5)
Zn1—S3	2.6080 (10)	C10—H10	0.9300
Zn1—S4	2.3669 (9)	C10—C11	1.297 (6)
Zn1—N3	2.103 (2)	C11—H11A	0.9300
S1—C1	1.725 (3)	C11—H11B	0.9300
S2—C1	1.716 (3)	C12—H12A	0.9700
S3—C8	1.715 (3)	C12—H12B	0.9700
S4—C8	1.722 (3)	C12—C13	1.495 (5)
N1—C1	1.331 (4)	C13—H13	0.9300
N1—C2	1.473 (4)	C13—C14	1.277 (5)
N1—C5	1.480 (4)	C14—H14A	0.9300
N2—C8	1.338 (4)	C14—H14B	0.9300
N2—C9	1.465 (4)	C15—C16	1.403 (4)
N2—C12	1.466 (4)	C15—C20	1.417 (4)
N3—C15	1.386 (4)	C16—H16	0.9300
N3—C23	1.340 (4)	C16—C17	1.373 (4)
C2—H2A	0.9700	C17—H17	0.9300
C2—H2B	0.9700	C17—C18	1.405 (5)
C2—C3	1.486 (5)	C18—H18	0.9300
C3—H3	0.9300	C18—C19	1.356 (5)
C3—C4	1.292 (5)	C19—H19	0.9300
C4—H4A	0.9300	C19—C20	1.413 (4)
C4—H4B	0.9300	C20—C21	1.410 (4)
C5—H5A	0.9700	C21—H21	0.9300
C5—H5B	0.9700	C21—C22	1.354 (4)
C5—C6	1.470 (5)	C22—H22	0.9300
C6—H6	0.9300	C22—C23	1.407 (4)
C6—C7	1.272 (6)	C23—C24	1.501 (4)
C7—H7A	0.9300	C24—H24A	0.9600
C7—H7B	0.9300	C24—H24B	0.9600
C9—H9A	0.9700	C24—H24C	0.9600
			
S1—Zn1—S2	73.21 (3)	N2—C9—C10	113.7 (3)
S1—Zn1—S3	98.65 (3)	H9A—C9—H9B	107.7
S2—Zn1—S3	167.33 (3)	C10—C9—H9A	108.8
S4—Zn1—S1	118.16 (3)	C10—C9—H9B	108.8
S4—Zn1—S2	102.45 (3)	C9—C10—H10	116.8
S4—Zn1—S3	72.44 (3)	C11—C10—C9	126.4 (4)
N3—Zn1—S1	122.15 (7)	C11—C10—H10	116.8
N3—Zn1—S2	98.16 (7)	C10—C11—H11A	120.0
N3—Zn1—S3	94.40 (7)	C10—C11—H11B	120.0
N3—Zn1—S4	119.53 (7)	H11A—C11—H11B	120.0
C1—S1—Zn1	86.86 (11)	N2—C12—H12A	109.3
C1—S2—Zn1	82.25 (11)	N2—C12—H12B	109.3
C8—S3—Zn1	80.86 (12)	N2—C12—C13	111.5 (3)
C8—S4—Zn1	88.28 (11)	H12A—C12—H12B	108.0
C1—N1—C2	123.5 (3)	C13—C12—H12A	109.3
C1—N1—C5	122.3 (3)	C13—C12—H12B	109.3
C2—N1—C5	114.1 (3)	C12—C13—H13	116.6
C8—N2—C9	121.6 (3)	C14—C13—C12	126.7 (4)
C8—N2—C12	122.4 (3)	C14—C13—H13	116.6
C9—N2—C12	115.9 (3)	C13—C14—H14A	120.0
C15—N3—Zn1	120.54 (18)	C13—C14—H14B	120.0
C23—N3—Zn1	120.4 (2)	H14A—C14—H14B	120.0
C23—N3—C15	118.8 (3)	N3—C15—C16	120.2 (3)
S2—C1—S1	117.62 (19)	N3—C15—C20	121.1 (3)
N1—C1—S1	121.0 (2)	C16—C15—C20	118.6 (3)
N1—C1—S2	121.3 (2)	C15—C16—H16	119.7
N1—C2—H2A	109.3	C17—C16—C15	120.5 (3)
N1—C2—H2B	109.3	C17—C16—H16	119.7
N1—C2—C3	111.6 (3)	C16—C17—H17	119.6
H2A—C2—H2B	108.0	C16—C17—C18	120.9 (3)
C3—C2—H2A	109.3	C18—C17—H17	119.5
C3—C2—H2B	109.3	C17—C18—H18	120.2
C2—C3—H3	118.0	C19—C18—C17	119.6 (3)
C4—C3—C2	123.9 (4)	C19—C18—H18	120.2
C4—C3—H3	118.0	C18—C19—H19	119.5
C3—C4—H4A	120.0	C18—C19—C20	121.0 (3)
C3—C4—H4B	120.0	C20—C19—H19	119.5
H4A—C4—H4B	120.0	C19—C20—C15	119.3 (3)
N1—C5—H5A	109.6	C21—C20—C15	118.0 (3)
N1—C5—H5B	109.6	C21—C20—C19	122.7 (3)
H5A—C5—H5B	108.1	C20—C21—H21	120.1
C6—C5—N1	110.5 (3)	C22—C21—C20	119.8 (3)
C6—C5—H5A	109.6	C22—C21—H21	120.1
C6—C5—H5B	109.6	C21—C22—H22	119.9
C5—C6—H6	116.7	C21—C22—C23	120.2 (3)
C7—C6—C5	126.5 (4)	C23—C22—H22	119.9
C7—C6—H6	116.7	N3—C23—C22	121.9 (3)
C6—C7—H7A	120.0	N3—C23—C24	119.1 (3)
C6—C7—H7B	120.0	C22—C23—C24	119.0 (3)
H7A—C7—H7B	120.0	C23—C24—H24A	109.5
S3—C8—S4	118.01 (19)	C23—C24—H24B	109.5
N2—C8—S3	121.1 (2)	C23—C24—H24C	109.5
N2—C8—S4	120.9 (2)	H24A—C24—H24B	109.5
N2—C9—H9A	108.8	H24A—C24—H24C	109.5
N2—C9—H9B	108.8	H24B—C24—H24C	109.5
			
Zn1—S1—C1—S2	−2.37 (15)	C5—N1—C2—C3	74.9 (4)
Zn1—S1—C1—N1	176.3 (2)	C8—N2—C9—C10	96.1 (4)
Zn1—S2—C1—S1	2.24 (15)	C8—N2—C12—C13	84.4 (4)
Zn1—S2—C1—N1	−176.5 (3)	C9—N2—C8—S3	179.9 (2)
Zn1—S3—C8—S4	5.83 (15)	C9—N2—C8—S4	0.2 (4)
Zn1—S3—C8—N2	−173.9 (3)	C9—N2—C12—C13	−93.9 (4)
Zn1—S4—C8—S3	−6.35 (16)	C12—N2—C8—S3	1.7 (4)
Zn1—S4—C8—N2	173.3 (3)	C12—N2—C8—S4	−178.0 (2)
Zn1—N3—C15—C16	−9.7 (4)	C12—N2—C9—C10	−85.6 (4)
Zn1—N3—C15—C20	170.7 (2)	C15—N3—C23—C22	3.6 (4)
Zn1—N3—C23—C22	−170.1 (2)	C15—N3—C23—C24	−177.4 (3)
Zn1—N3—C23—C24	8.9 (4)	C15—C16—C17—C18	−0.9 (5)
N1—C2—C3—C4	−119.1 (4)	C15—C20—C21—C22	1.2 (5)
N1—C5—C6—C7	−115.3 (4)	C16—C15—C20—C19	0.1 (5)
N2—C9—C10—C11	−3.0 (6)	C16—C15—C20—C21	−179.0 (3)
N2—C12—C13—C14	108.5 (5)	C16—C17—C18—C19	0.3 (6)
N3—C15—C16—C17	−178.9 (3)	C17—C18—C19—C20	0.4 (6)
N3—C15—C20—C19	179.7 (3)	C18—C19—C20—C15	−0.7 (5)
N3—C15—C20—C21	0.6 (5)	C18—C19—C20—C21	178.4 (3)
C1—N1—C2—C3	−108.6 (4)	C19—C20—C21—C22	−177.9 (3)
C1—N1—C5—C6	−91.1 (4)	C20—C15—C16—C17	0.7 (5)
C2—N1—C1—S1	7.2 (4)	C20—C21—C22—C23	−0.6 (5)
C2—N1—C1—S2	−174.2 (2)	C21—C22—C23—N3	−1.9 (5)
C2—N1—C5—C6	85.4 (4)	C21—C22—C23—C24	179.2 (3)
C5—N1—C1—S1	−176.6 (2)	C23—N3—C15—C16	176.6 (3)
C5—N1—C1—S2	2.0 (4)	C23—N3—C15—C20	−3.0 (4)

### [Bis(prop-2-en-1-yl)carbamodithioato-κ2S,S'](2-methylquinoline-κN)zinc(II) Hydrogen-bond geometry (Å, º)

**Table:**

*D*—H···*A*	*D*—H	H···*A*	*D*···*A*	*D*—H···*A*
C2—H2*B*···S1	0.97	2.55	3.048 (4)	112
C9—H9*A*···S4	0.97	2.52	2.999 (4)	110
C16—H16···S1	0.93	2.88	3.742 (3)	155
C24—H24*A*···S4	0.96	2.78	3.694 (3)	158
C24—H24*C*···S2	0.96	2.90	3.505 (4)	122
